# The role of the miR-30a-5p/BCL2L11 pathway in rosmarinic acid-induced apoptosis in MDA-MB-231-derived breast cancer stem-like cells

**DOI:** 10.3389/fphar.2024.1445034

**Published:** 2024-08-22

**Authors:** Wei Wang, Yuefen Zhang, Xiaomin Huang, Dan Li, Qi Lin, Hailin Zhuang, Hong Li

**Affiliations:** ^1^ School of Public Health and Health Management, Fujian Health College, Fuzhou, Fujian, China; ^2^ Science and Technology Service Center, Fujian Health College, Fuzhou, Fujian, China; ^3^ School of Pharmacy, Fujian Health College, Fuzhou, Fujian, China

**Keywords:** apoptosis, miR-30a-5p, BCL2L11, BCSCs, rosmarinic acid, triple-negative breast cancer

## Abstract

**Background:**

Rosmarinic acid (RA), a natural phenolic acid, exhibits promising anti-cancer properties. The abnormal expression of microRNA (miRNA) regulates the gene expression and plays a role as an oncogenic or tumor suppressor in TNBC. However, the biological role of RA in miR-30a-5p on BCL2L11 during MDA-MB-231 induced breast cancer stem-like cells (BCSCs) progression and its regulatory mechanism have not been elucidated.

**Objective:**

To investigate whether RA inhibited the silencing effect of miR-30a-5p on the BCL2L11 gene and promoted apoptosis in BCSCs.

**Materials and Methods:**

We assessed the migration, colony formation, proliferation, cell cycle, and apoptosis of BCSCs after RA treatment using the wound-healing assay, colony formation assay, CCK-8 assay, and flow cytometry, respectively. The expression of mRNA and protein levels of BCL-2, Bax, BCL2L11, and P53 genes in BCSCs after RA treatment was obtained by real-time polymerase chain reaction and Western blot. Differential miRNA expression in BCSCs was analyzed by high-throughput sequencing. Targetscan was utilized to predict the targets of miR-30a-5p. The dual luciferase reporter system was used for validation of the miR-30a-5p target.

**Results:**

Wound-healing assay, colony formation assay, CCK-8 assay, and cell cycle assay results showed that RA inhibited migration, colony formation and viability of BCSCs, and cell cycle arrest in the G0-G1 phase. At the highest dose of RA, we noticed cell atrophy, while the arrest rate at 100 μg/mL RA surpassed that at 200 μg/mL RA. Apoptotic cells appeared early (Membrane Associated Protein V FITC^+^, PI^−^) or late (Membrane Associated Protein V FITC^+^, PI^+^) upon administration of 200 μg/mL RA, Using high-throughput sequencing to compare the differences in miRNA expression, we detected downregulation of miR-30a-5p expression, and the results of dual luciferase reporter gene analysis indicated that BCL2L11 was a direct target of miR-30a-5p.

**Conclusion:**

RA inhibited the silencing effect of miR-30a-5p on the BCL2L11 gene and enhanced apoptosis in BCSCs.

## Graphical Abstract

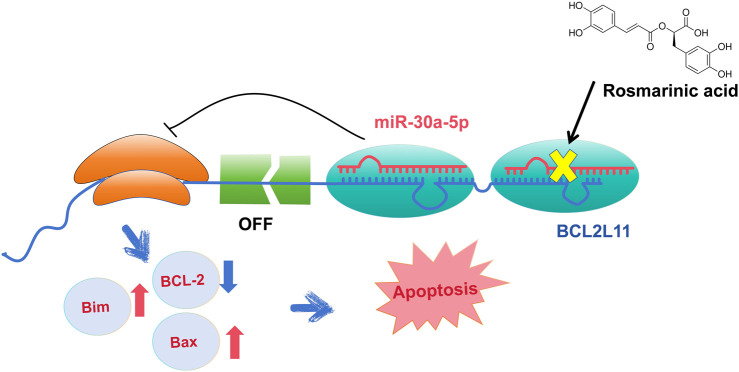

## Highlight

• MicroRNAs (miRNAs) is involved in the development and development of various cancers, including breast cancer. In this study, we explored the impact of miR-30a-5p on cell viability, migration, colony integration, and cell cycle in BCSCs after RA treatment, and we discovered that miR-30a-5p directly targets BCL2L11. These findings broaden and deepen the understanding of the molecular functions of miR-30a-5p and BCL2L11.

## 1 Introduction

Breast cancer is one of the most common malignancies in women. The latest results released by the World Health Organization show that about 2.3 million new cases have occurred in 2022, accounting for 11.7% of all cancer cases. It is the fourth leading cause of cancer death worldwide, with approximately 670,000 deaths, representing 6.9% of all cancer deaths (Organization, 2024). Compared to estrogen receptor-positive breast cancer, triple-negative breast cancer (TNBC) is characterized by the absence of estrogen receptor, progesterone receptor, and human epidermal growth factor receptor-2. It is also characterized by high invasion, poor prognosis, high recurrence rate, and high mortality, accounting for about 15%–20% of all breast cancer pathological types (Ueda et al., 2020; Yin et al., 2020), and the 5-year survival rate of TNBC patients is less than 30% (Yin et al., 2020). The current treatment options for TNBC encompass diverse modalities including surgery, chemotherapy, radiotherapy, and drug therapy. Rosmarinic acid (RA) offers a selective therapeutic pathway. Our Previous study conducted cytotoxicity assessments, revealing that RA exhibits significantly higher inhibitory effects on TNBC tumor cells compared to normal breast cells (e.g., MCF-10A) (Ni et al., 2019).

Recent studies have indicated that breast cancer stem cell-like stem cells (BCSCs) and BCSCs represent a small number of subsets of cancer cells. These cells are characterized by a CD44^+^/CD24^-/low^ phenotype and are known to exhibit longevity, high proliferation, self-renewal potential, plasticity, chemotherapy resistance, and apoptosis resistance (Al-Hajj and Clarke, 2004). In the previous study of our research group, we have successfully sorted out the BCSCs with the characteristics of the CD44^+^/CD24^-/low^ phenotype (Ni et al., 2019). However, the effect of drugs on BCSCs in breast cancer is relatively weak, and moreover, is closely related to the patient’s prognosis (Liu et al., 2018).

Sarcandra glabra (S. glabra) has a long history of clinical applications in treating rheumatism and pain, and falling and beating injuries (Zeng et al., 2021). Recent studies have found that its extract is effective in various tumor treatments. One notable example is the significant improvement in chemotherapy efficacy for patients with advanced esophageal cancer when using arcandra glabra injection (Dong et al., 2019). In the extract of S. glabra, rosmarinic acid (RA) stands out as one of the phenolic acids with the highest content, serving as a quality control marker in the Chinese Pharmacopoeia for S. glabra (Zeng et al., 2021). In addition, recent studies have found that RA has anti-inflammatory, antibacterial, anti-viral, anti-oxidation, anti-tumor and other pharmacological effects (Nunes et al., 2017; Guan et al., 2022; Zhao et al., 2022; Ijaz et al., 2023). In a previous study, we isolated BCSCs from the MDA-MB-231 cell line and demonstrated that RA inhibited the viability and migration of BCSCs and induced apoptosis. Our observed associated reductions in the BCL-2/Bax ratio and Hh signaling (i.e., reduced Smo and Gli 1 pathways) suggest that these pathways are useful targets for drug development f to treat TNBC (Ni et al., 2019).

The BCL-2 protein family plays a crucial role in apoptosis signaling pathway. Scientists categorize these proteins into anti-apoptotic and pro-apoptotic groups based on their different structural domains. BCL-2 is a representative of the anti-apoptotic proteins, while Bax represents the pro-apoptotic proteins. The protein expression product of the anti-apoptotic gene BCL-2 is an intracellular membrane protein, which is mainly localized in the mitochondrial, endoplasmic reticulum, and nuclear membranes of the cell, and exerts anti-apoptotic effects. Bax, a protein homologous to BCL-2, contains three BH domains, and has pro-apoptotic effects (Muller-Rover et al., 1999). The relative ratio of BCL-2/Bax is a critical link in the induction of apoptosis after receiving a stimulatory signal (Zhou et al., 2017; Hu et al., 2018). Significant reduction in BCL-2/Bax ratio have been observed in various tumor cell lines following RA treatment (Jang et al., 2018; Li et al., 2019). Whereas our previous findings showed increased apoptosis and downregulation of BCL-2/Bax ratio in MDA-MB-231 cell-derived BCSCs after RA treatment (Li et al., 2018a; Ren et al., 2018). However, the molecular mechanisms by which RA regulates BCL-2/Bax expression remain to be further explored.

Micro-RNAs (miRNAs) are a class of small non-coding RNAs of 19–25 nucleotides in length, which can regulate the gene expression by binding to the 3′untranslated region (3′UTR) of the target gene mRNA, leading to post-transcriptional repression or degradation (Winkler, 2010). Dysregulated tiny RNA may play a role in tumor suppression or promotion by binding oncogenes or tumor suppressor genes (Zheng and Zhang, 2016). It has also been preliminarily reported that RA suppresses the proliferation of tumor cells through the regulation of miRNA. For example, RA restored the sensitivity of drug-resistant gastric cancer cell lines to 5-fluorouracil by inhibiting miR-6785-5p and miR-642a-3p (Yu et al., 2019). This demonstrates that phenolic acids in plants can regulate the expression of relevant genes through miRNA mechanism and thus inhibit TNBC proliferation (Kilic et al., 2019). However, it is unclear whether RA affects the development of MDA-MB-231 cell-derived BCSCs through miRNA.

Increasing studies have shown that miR-30a-5p is altered in a variety of tumors, such as colorectal cancer (Wei et al., 2016), non-small cell lung cancer (Zhu et al., 2017), and hepatocellular carcinoma (He et al., 2015), especially in breast cancer (Li L. et al., 2017). However, the role of miR-30a-5p in TNBC after RA action is currently unknown.

Therefore, further studies are needed to determine the regulatory role of miR-30a-5p in RA in BCSCs. We hypothesized that RA could induce apoptosis by altering miR-30a-5p levels in BCSCs and affecting the downstream expression of the BCL2L11 gene and related apoptotic genes (e.g., BCL-2, Bax, P53).

## 2 Materials and methods

### 2.1 Materials

The purity of RA extracted from S. glabra was more than 98% (identified by UPLC, Saychun Biotechnology Co. Ltd., Hubei, China). RA was dissolved in 0.1% dimethyl sulfoxide (DMSO) at the final concentrations of 0, 50, 100 and 200 μg/mL.

### 2.2 Cell culture and BCSCs enrichment

BCSCs were seeded in RPMI-1640 medium (HyClone; cat. #SH30809.01) and 100 ng/mL cholera toxin. All the mediums were supplemented with 10% fetal calf serum (PAN Biotech, Aidenbach, German) in the presence of streptomycin and penicillin. BCSCs were enzymatically lysed in 0.05% trypsin/0.025% EDTA solution and cell suspensions were cultured in bottles with low surface attachment (Corning) in serum-free RPMI-1640 medium containing 10 ng/mL basic fibroblast growth factor (Peprotech, Rocky Hill, NJ; cat. #AF-100-15), 20 ng/mL EGF (Peprotech, Rocky Hill, NJ; cat. #100-18B) and 2% B-27 Supplement (Gibco, New York, United States; cat. #17504-044). Such culture conditions help the cell suspensions convert to sphere-forming cells. All these cells were incubated in a humidified 5% CO_2_ incubator at 37°C.For transfection experiments, miR-30a-5p mimic was obtained from Sangon Biotech (Shanghai, China). Then, the cells were seeded in 6-well plates and transfected according to the supplier′s instructions. The sequences of miR-30a-5p mimic as follows:sense (5′-3′):UGU​AAA​CAU​CCU​CGA​CUG​GAA​G; antisense (5′-3′):UCC​AGU​CGA​GGA​UGU​UUA​CAU​U.

### 2.3 Wound healing assay for cell migration analysis

BCSCs were seeded at a density of 2.5 × 10^5^ cells per well in 6-well culture plates and allowed to form a confluent monolayer. The layer of cells was scraped with a 200 μL micropipette tip to create a wound. Cells were washed twice with PBS and replaced with 10% serum medium containing various concentrations of 0, 50, 100 and 200 μg/mL of RA at 0, 24, 48, and 72 h (3 wells per group), the width of the wound (3 wounds per well) was monitored under a phase-contrast microscope at × 100 and measured using an image analysis system (Image-Pro Plus 5.0; Media Cybernetics).

### 2.4 Colony-formation assay

Colony formation analysis determined the effect of 0, 50, 100 and 200 μg/mL of RA on BCSCs at 0, 24, 48, and 72 h. Briefly, 500 cells were cultured in 6-well plates containing 1 mL DMEM and supplemented with 10% FBS. The culture medium was changed once every 3 days. After 2 weeks, the medium was discarded, and the cells were fixed with 4% paraformaldehyde and stained with crystal violet for 30 min. Colonies and lithography were counted.

### 2.5 Cell apoptosis detection

5 × 10^5^ BCSCs were harvested and incubated with different concentrations of 0, 100 and 200 μg/mL RA for 48 h (3 wells per group). Cells were washed once with cold PBS, resuspended in 1 mL PBS, then dual stained with 5 μL of Annexin V-FITC and 10 μL of PI, and then incubated in the dark for 30 min. Flow Cytometry was carried out to identify apoptotic populations of the BCSCs using the FACSVerse (BD Biosciences, United States).

### 2.6 Cell viability assay

Cell viability was determined using the CCK-8 assay (BeyotimeInst Biotech, China). Briefly, 1 × 10^4^ BCSCs in each well were treated with different concentrations of 0, 100 and 200 μg/mL RA for 0, 24, 48, or 72 h. 10 μL CCK-8 solution was added to each well and incubated at 37°C for 3 h, and the absorbance was determined at 450 nm from 5 replicates using a microplate reader Bio-RAD 680 (United States). Densitometric analysis was performed, and the levels of RA-treated cells were normalized against the levels of the DMSO vehicle group. Each experiment was independently replicated at least three times.

### 2.7 Cell cycle assay

To determine the effect of 0, 100 and 200 μg/mL RA on the cell cycle distribution of BCSCs, we performed cell cycle analysis using flow cytometry. Cells were treated at 60%–70% confluency in a T-75 flask. Each experiment was done in triplicate. Briefly, following sample treatment and incubation, cells were harvested, washed, and fixed with absolute ethanol and stored until ready for use. Samples were vortexed and then centrifuged at 3,000 rpm for 5 min. Ethanol was removed, and cells were resuspended in a staining buffer (PBS with 25 μg/mL RNase A, 50 μg/mL Propidium Iodide). Stained cells were incubated at room temperature (30–60 min). FACSC calibur flow cytometer (B.D. Biosciences, San Jose, CA, United States) was used to determine the proportion of cells in each cell cycle stage within 2 h of staining. Before the analysis, the instrument was aligned with Calibrite beads (B.D. Biosciences). Samples were further analyzed using the ModFit LT 6.0 software (Verity Software House, Bedford, MA, United States).

### 2.8 RNA isolation and quantification

The mRNA levels were determined using reverse transcriptase polymerase chain reaction (RT-PCR). After the BCSCs (3 wells of a 6-well plate per group) were treated with different concentrations of 0, 100 and 200 μg/mL RA for 48 h, total RNA was extracted using the TRIzol reagent (Takara Inc., Dalian, China; cat. #9109) and then reverse-transcribed to complementary DNA (cDNA) using the RNase Hi (Takara Inc., Dalian, China; cat. #RR037A). Real-time quantitative PCR was then conducted using the SYBR Green I fluorescent dye reagent (Roche Inc., Shanghai, China; cat. #19317900) with the ABI System Sequence Detector 7500. β-actin was used as an internal standard. PCR amplifications were performed for all samples under the following conditions: (stage 1, 1 cycle) 95°C for 30 s; (stage 2, 40 cycles) 95°C for 5 s, 60°C for 32 s. For each sample, a triplicate of PCR experiments was conducted and averaged to eliminate loading errors. β-actin is calculated as an internal standard to calculate the relative amounts (2^−ΔΔCT^) of the mRNA of genes of interest. Primer sequences (Sangon Biotech, Shanghai, China) viewed in Table 1.

**TABLE 1:** Primer sequences used in this study.

Genes	Forward primer (5′-3′)	Reverse primer (5′-3′)
β-actin	CAT​GTA​CGT​TGC​TAT​CCA​GGC	CTC​CTT​AAT​GTC​ACG​CAC​GAT
BCL-2	GTG​TGT​GGA​GAG​CGT​CAA​CC	AGA​AAT​CAA​ACA​GAG​GCC​GCA
Bax	AGC​GAC​TGA​TGT​CCC​TGT​CT	TCC​AGA​TGG​TGA​GTG​AGG​CG
BCL2L11	GCC​TTC​AAC​CAC​TAT​CTC​AG	TAA​GCG​TTA​AAC​TCG​TCT​CC
P53	CTC​TCC​CAC​CAA​CAT​CCA​CT	ACGTCCACC ACCATTTGAAC

### 2.9 Western-blot assay

After the BCSCs (3 wells per group) were treated with different concentrations of RA for 48 h, the total protein of the BCSCs was extracted using BCA kit (Beyotime, China). Protein samples containing equal amounts of protein (40 μg) were size-fractionated by electrophoresis and proteins were transferred from a gel to a polyvinylidene fluoride (PVDF) membrane. The PVDF membrane was then incubated with Tris-buffered saline (pH 7.5) and 5% skim milk for 1 h at 37°C to block the binding of nonspecific proteins. The PVDF membranes were then 4°C with primary antibodies specific to BCL-2 (1:1,000, Huabio Inc., China, cat. #ET1603-11), Bax (1:1,000, Huabio Inc., China, cat. #ET1603-34), BCL2L11 (1:1,000, Huabio Inc., China, cat. #ET1608-04) and P53 (1:1,000, Huabio Inc., China, cat. #ET1601-13). The membranes were washed in TBST, incubated with secondary HRP-linked antibodies (1:5,000, Huabio Inc., China, cat. #HA1006), and then imaged with the Gel Imaging System (Clinx, China). The relative levels of proteins of interest were calculated after normalized to the β-actin levels that serve as loading controls.

### 2.10 miRNA sequencing

miRNA sequencing was implemented by Illumina HiSeq (KangChen Bio-tech, Shanghai, China). In brief, total RNA was quantified with a NanoDrop ND-100. Small RNA adapters were then ligated to the 5′and 3′ends of total RNA. After cDNA synthesis and amplification, the PCR-amplified fragments were purified from the PAGE gel, and the completed cDNA libraries were quantified by an Agilent 2,100 Bioanalyzer. Cluster generation was performed on an Illumina cBot, and sequencing was performed on an Illumina HiSeq 2000 according to the manufacturer’s instructions.miRNAs with FC > 2.0 and *p* < 0.05 were considered to be differentially expressed.

### 2.11 Dual-luciferase reporter assay

The method is based on the PAS (PCR-based Accurate Synthesis), to design the full-length overlap primers, The sequence of BCL2L11(NM 138621.5)-WT (wild type), BCL2L11 (NM 138621.5)-MUT (mutant) were synthesized by PCR, Separtively into the psicheck2.0 vector, The BCL2L11 (NM 138621.5)-WT and BCL2L11 (NM 138621.5)-MUT recombinant vector, Meanwhile, hsa-miR-30a-5p mimics was synthesized *in vitro*; Cotransferred into BCSCs by Lipo3000 liposome transfection, By using a dual-luciferase assay kit (DD1205, NovPro) to detect the regulatory effect of miR-30a-5p on BCL2L11 gene. Relative luciferase activity was calculated by dividing the firefly luciferase assay results by the Renilla luciferase assay results.

### 2.12 Statistical analysis

Data are expressed as mean ± standard deviation (SD). Differences between multiple groups were analyzed by one-way analysis of variance (ANOVA), followed by the Tukey test. The Student’s t-test was used to assess the significant differences between the two groups. Statistical analysis was performed using GraphPad (version 6.0) and SPSS (version 25.0, SPSS, IBM, Inc., Armonk, NY, United States). Alpha was set at 0.05. All the experiments were repeated three times.

## 3 Results

### 3.1 RA inhibited the migration in BCSCs

**FIGURE 1:**
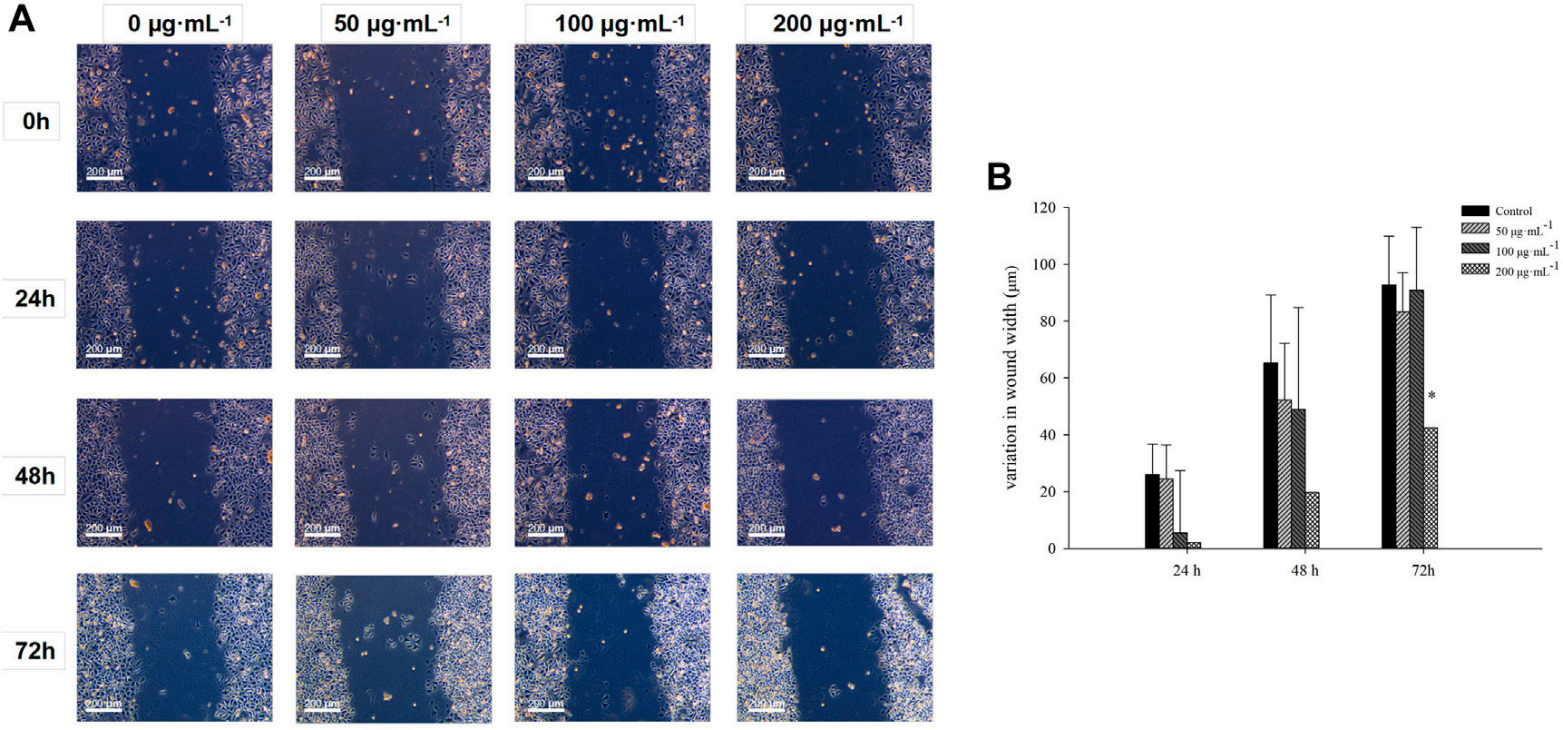
Comparison of the inhibitory effects of BCSCs on wound healing inhibition after RA treatment. **(A, B)** Variation of wound width in cultured BCSCs after 0, 50, 100 and 200 μg/mL RA treatment for 0, 24, 48 and 72 h * indicates *P* < 0.05, compared with control. group n = 3.

Wound healing assay was performed on cultured cells to investigate how RA affects BCSCs migration; mechanical removal of the cell culture center was positively correlated with the rate of cell migration after dissection (Figure 1A). The migration of BCSCs was effectively blocked with 200 μg/mL RA at 0, 24, 48, and 72 h, compared to control (*P* < 0.05) (Figure 1B). Note that cell size shrinkage of BCSCs was observed after RA treatment with the highest concentration (200 μg/mL) (Figure 1A).

### 3.2 RA inhibited colony formation in BCSCs

**FIGURE 2:**
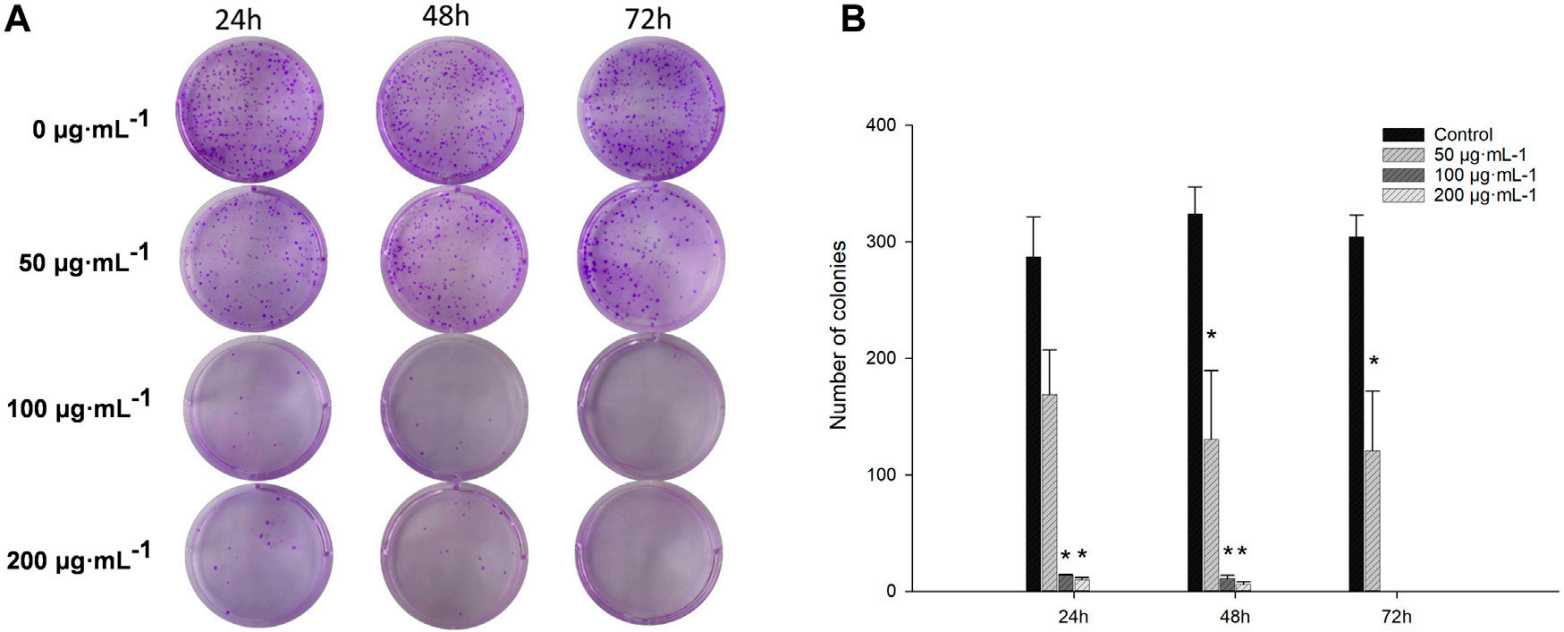
Comparison of colony formation in BCSCs after RA treatment. **(A, B)** Changes in colony formation size of cultured BCSCs treated at 0, 50, 100 and 200 μg/mL RA for 24, 48, and 72 h* indicates *P* < 0.05, compared with control. group n = 3.

Cell colony assays on cultured cells to investigate the effect of RA on colony formation in BCSCs showed that 100–200 μg/mL RA effectively inhibited colony formation in BCSCs at 24, 48, and 72 h compared to the control group. No significant difference was seen with 50 μg/mL RA treatment at 24 h compared to the control, and when RA treatment was extended to 48 and 72 h, 50 μg/mL RA also effectively inhibited the colony formation in BCSCs (Figures 2A, B).

### 3.3 RA decreased the viability and blocked the cell cycle in BCSCs

**FIGURE 3:**
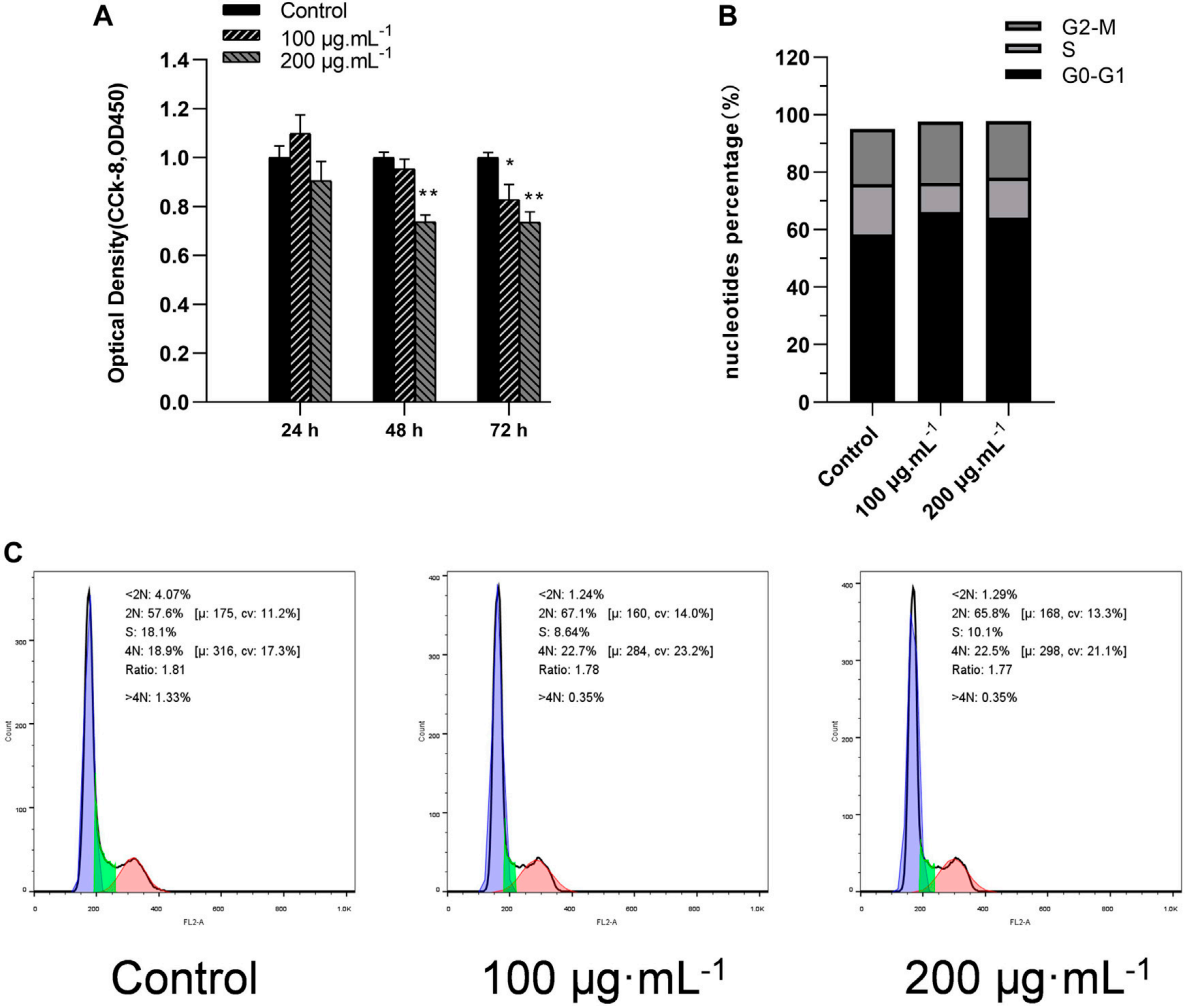
Effect of BCSCs on cell viability and cell cycle after RA treatment. **(A)** Cell viability of BCSCs treated at 0, 100 and 200 μg/mL RA for 24, 48, and 72 h by CCK-8 assay. The DMSO was used as the control group. **(B)** Percent cell cycle results of cultured BCSCs treated at 0, 100 and 200 μg/mL RA for 48 h. **(C)** Apoptotic populations of the BCSCs treated at 0, 100 and 200 μg/mL RA for 48 h were identified by flow cytometry. * and ** indicate *P* < 0.05, 0.01, compared with control. group n = 3.

The effect of RA on BCSCs viability was assessed by CCK-8 assay. At 48 h, only 200 μg/mL RA significantly inhibited cell viability (all *P* < 0.05). And when extending the duration of RA action to 72 h, lower doses of 100 μg/mL also showed a significant effect of inhibiting cancer cell viability (all *P* < 0.05) (Figure 3A). Cell cycle assays on cultured cells to investigate the effect of RA on the cell cycle of BCSCs, BCSCs treated with 0.100 and 200 μg/mL RA for 48 h and analyzed using flow cytometry. The results showed that 100 and 200 μg/mL RA treatment increased both G0-G1 and S in BCSCs, with 100 μg/mL G0-G1 accumulation higher than 200 μg/mL (66.1% vs 64.2%) (Figures 3B, C).

### 3.4 RA induced apoptosis in BCSCs

**FIGURE 4:**
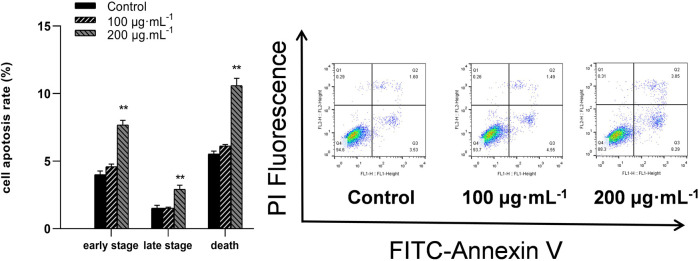
RA treatment causes apoptosis in BCSCs. Flow cytometry was performed to detect apoptotic cells treated with 0, 100 and 200 μg/mL RA for 48 h ** indicates *P* < 0.01, compared with control. group n = 3.

Flow cytometry assessed the apoptosis in BCSCs after 48 h of RA treatment by staining for annexin V-FITC/PI; FITC-conjugated annexin V labeled the externalization of phosphatidylserine in apoptotic cells, while PI stained the nuclei of all cells, including healthy and dead cells. Apoptotic cells were identified as early (Membrane Associated Protein V FITC^+^, PI^−^) or late (Membrane Associated Protein V FITC^+^, PI^+^) upon administration of 200 μg/mL RA (Figure 4).

### 3.5 RA reduced BCL2L11 expression in BCSCs

**FIGURE 5:**
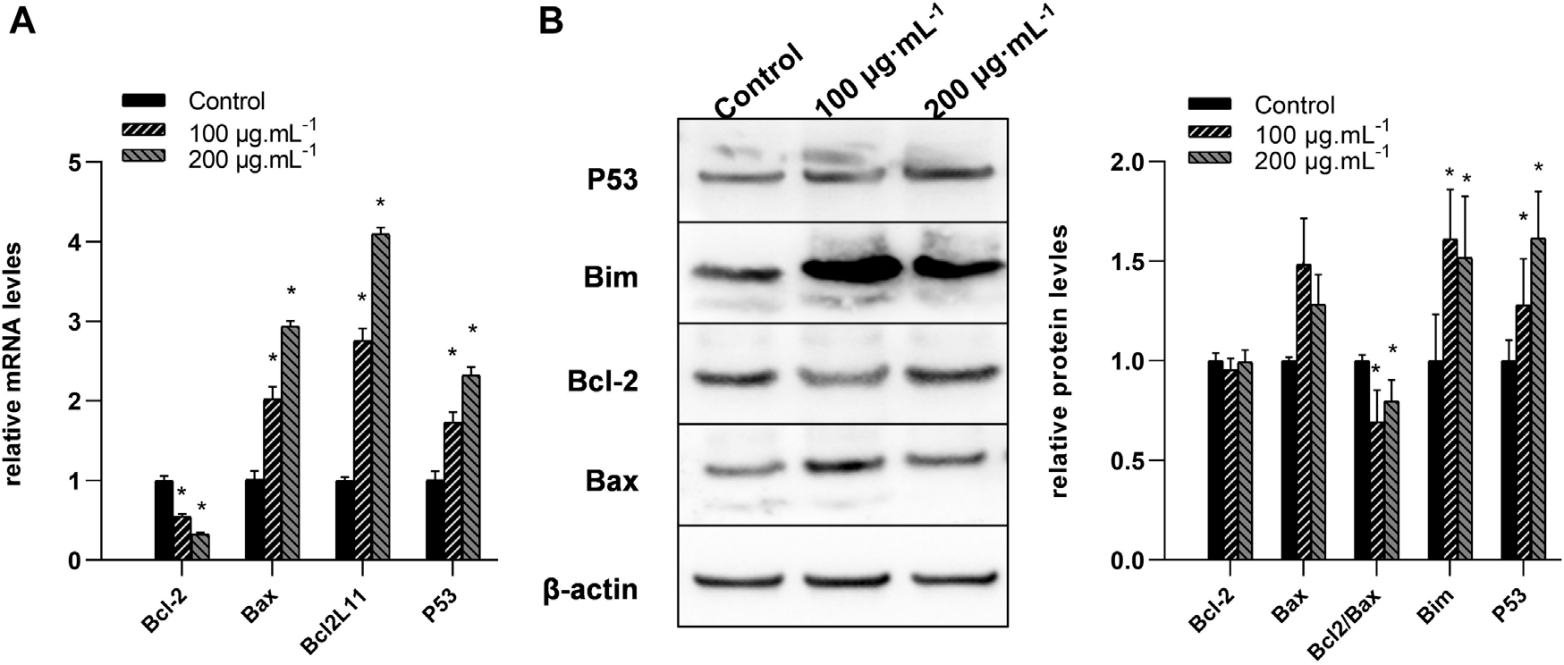
Changes of the mRNA and protein levels of BCL-2, Bax, BCL2L11, and P53 genes in BCSCs after RA treatment. **(A)** Quantitative reverse transcriptase polymerase chain reaction was used to compare the changes of the mRNA levels of BCL-2, Bax, BCL2L11, and P53 genes after 0, 100, and 200 μg/mL RA treatment at 48 h. **(B)** Left, western blot analysis of BCL-2, Bax, BCL2L11, and P53 proteins in the BCSCs; right, quantification of BCL-2, Bax, BCL2L11, and P53 proteins in the BCSCs, and data presented as bar representing mean value and error bars showing standard deviation. * indicates *P* < 0.05, compared with control. group n = 3.

Real-time RT-PCR analysis was conducted to measure the mRNA levels of P53 BCL2L11, BCL-2, and Bax genes, and immunoblot analysis was carried out to measure their protein levels. Compared to the control, the 100 and 200 μg/mL RA treatment significantly reduced the mRNA levels of BCL-2 while increasing those of BCL2L11, P53, and Bax (all *P* < 0.05) (Figure 5A). The 100 and 200 μg/mL RA treatment significantly decreased the protein levels of the BCL-2/Bax ratio while increasing the protein levels of Bim and P53 (all *P* < 0.05) (Figure 5B).

### 3.6 RA reduced miR-30a-5p expression in BCSCs

**FIGURE 6:**
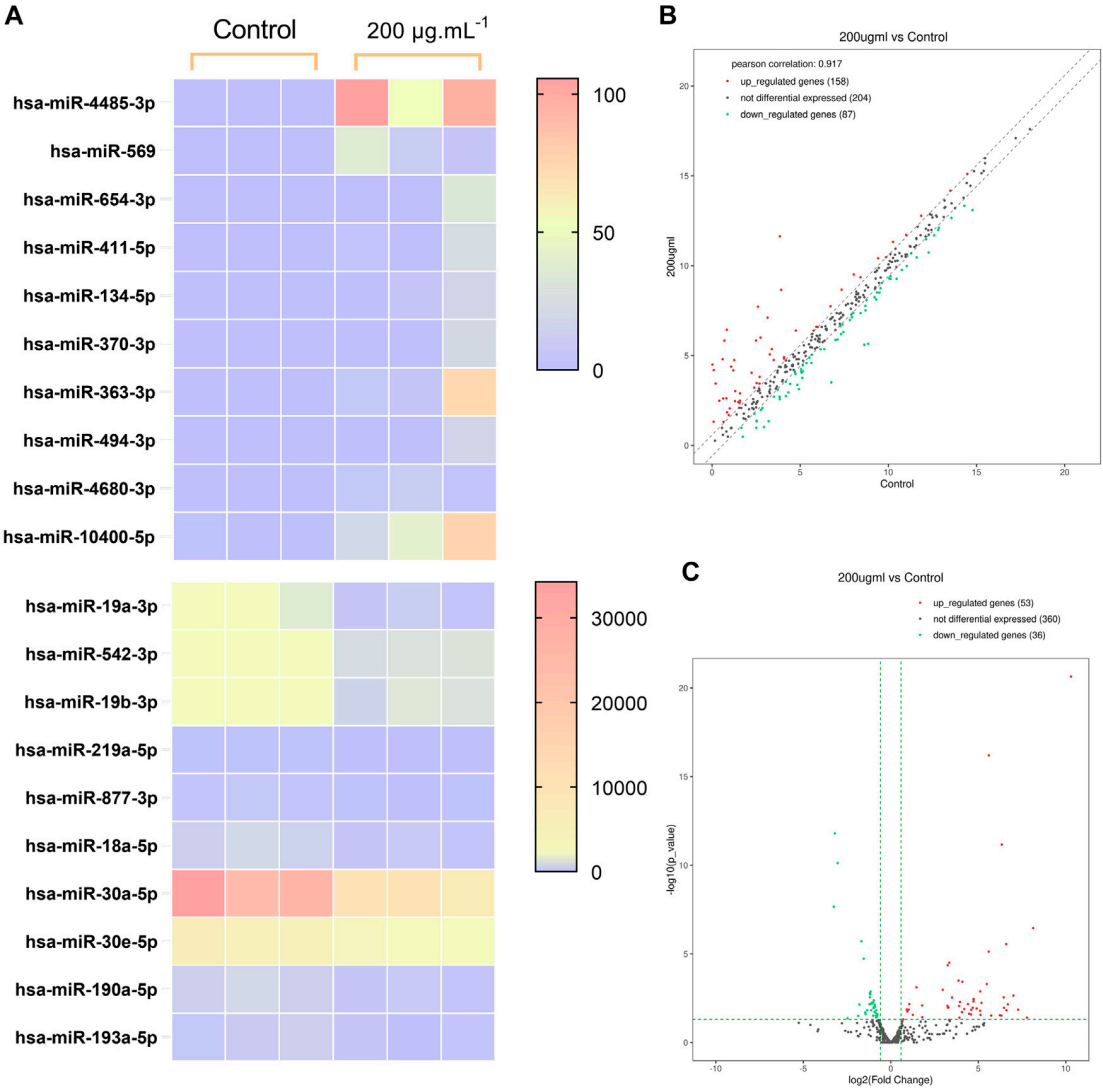
Differences in miRNA expression between the control group and BCSCs after 200 μg/mL RA treatment. **(A)** Heatmap of the clustering of the differentially expressed miRNA. **(B)** Scatterplot of the miRNA variants. **(C)** Volcano plot of the miRNA variation.

High throughput sequencing was used for the comparison of miRNA expression in BCSCs treated with 200 μg/mL RA and controls. We used high-throughput sequencing to compare miRNA expression in 200 μg/mL RA treated BCSCs and control groups, and detected 449 miRNAs. Among these, 32 miRNAs showed differential expression, with 53 upregulated and 36 downregulated. We present the first 10 up- and downregulated miRNAs in Table 2, where the Log2(FoldChange) of miR-30a-5p was −1.68. Differential miRNA expression is shown in the heat map, respectively (Figure 6A). Scatter plots and volcano plots illustrated the distribution and approximate number of miRNA (Figures 6B, C).

**TABLE 2:** Patterns of miRNA expression in BCSCs from 200 μg/mL RA treated and control groups.

miRNA	Log^2^(FoldChange)	*P*-value	Regulation^a^
hsa-miR-4485-3p	10.30041941	2.26188E-21	Up
hsa-miR-569	8.134568469	3.4713E-07	Up
hsa-miR-654-3p	7.278708182	0.01406	Up
hsa-miR-411-5p	7.006474478	0.00225	Up
hsa-miR-134-5p	6.687566358	0.00701	Up
hsa-miR-370-3p	6.612778726	0.01549	Up
hsa-miR-363-3p	6.598043286	2.83588E-06	Up
hsa-miR-494-3p	6.469883835	0.01093	Up
hsa-miR-4680-3p	6.450213044	0.00286	Up
hsa-miR-10400-5p	6.339730774	6.76648E-12	Up
hsa-miR-19a-3p	−3.255991474	2.20361E-08	Down
hsa-miR-542-3p	−3.19773582	1.57724E-12	Down
hsa-miR-19b-3p	−3.038521122	7.57063E-11	Down
hsa-miR-219a-5p	−2.472354636	0.04284	Down
hsa-miR-877-3p	−1.86317806	0.03185	Down
hsa-miR-18a-5p	−1.790663505	0.00732	Down
hsa-miR-30a-5p	−1.679151345	1.9247E-06	Down
hsa-miR-30e-5p	−1.54905592	0.00002	Down
hsa-miR-190a-5p	−1.465657913	0.02038	Down
hsa-miR-193a-5p	−1.437867637	0.02557	Down

^a^ 200 μg/mL vs. control.

### 3.7 GO and KEGG pathway Analyses

**FIGURE 7:**
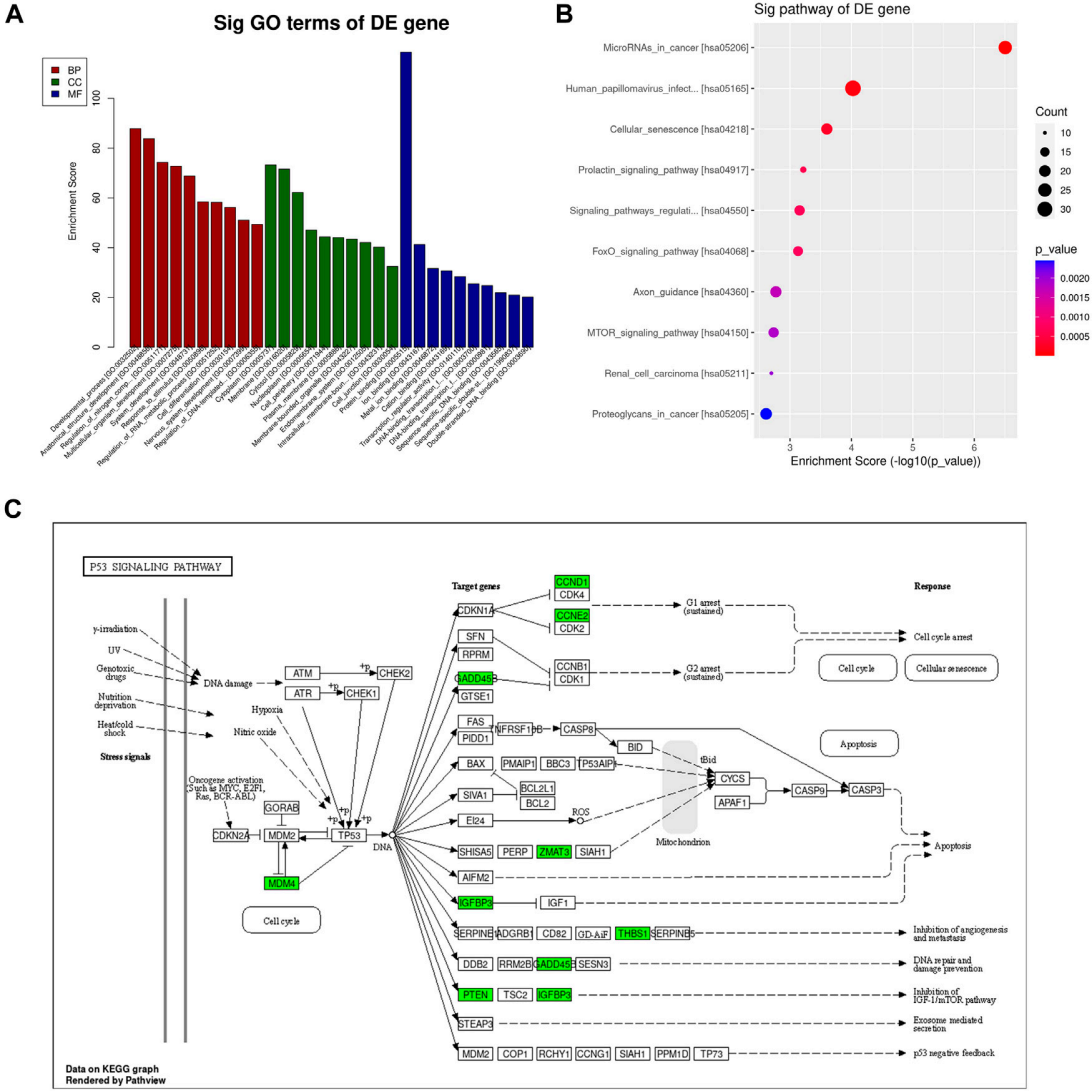
GO and KEGG pathway analysis of differentially expressed mRNAs. **(A)** The top 10 GO terms in biological process, cellular component, and molecular function are shown, ranked by enrichment score. **(B)** Top 10 pathways enriched among differentially regulated mRNAs. **(C)** P53 Pathway KEGG graph.

To clarify the biological functions of the genes *in vivo* or cells and the signaling pathways involved, we can annotate each gene based on the Gene Ontology and KEGG database. GO analysis showed that the differentially expressed miRNAs were mainly enriched in the developmental process, anatomical structure development, regulation of nitrogen compound metabolic process, multicellular organism development, system development, response to stimulus, regulation of RNA metabolic process, cell differentiation, nervous system development and regulation of DNA-templated transcription (Figure 7A). Pathway analysis showed that the differential miRNAs were mainly involved in miRNAs in cancer, Human papillomavirus infection, Cellular senescence, Prolactin signaling pathway, signaling pathways regulating pluripotency of stem cells, FoxO signaling pathway, Axon guidance, mTOR signaling pathway, Renal cell carcinoma and Proteoglycans in cancer (Figure 7B). The p53 signaling pathway is crucial in cancer incidence and progression and plays a significant role in the research on breast cancer suppression conducted in this study (Figure 7C).

### 3.8 miR-30a-5p binds to BCL2L11 and regulates apoptosis

**FIGURE 8:**
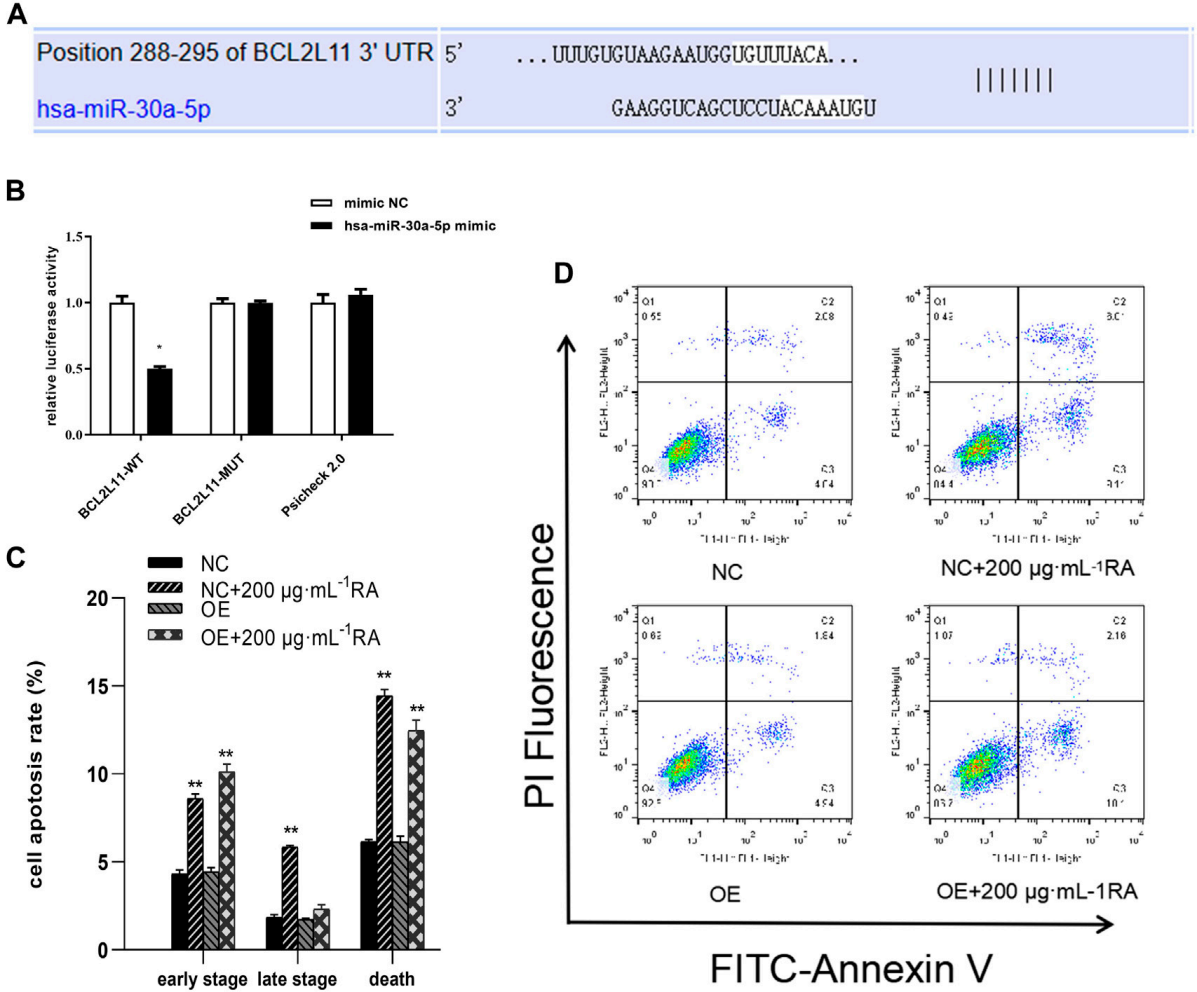
miR-30a-5p binds to BCL2L11 and regulates apoptosis. **(A)** TargetScan database predicted the existence of binding sites between BCL2L11 and miR-30a-5p. The mutation was generated on the BCL2L11 3′UTR sequence in the complementary site for the binding region of miR-30a-5p. **(B)** The luciferase reporter gene assay was performed using constructs containing predicted targeting sequences (BCL2L11-WT) and the mutated targeting sequence (BCL2L11-MUT). BCSCs were transfected with the constructs described above. **(C, D)** Apoptotic cells treated with 0 and 200 μg/mL RA for 48 h and/or overexpressing MiR-30a-5p were detected by flow cytometry. Results are presented as the mean ± SD. ** indicates *P* < 0.01, compared with control. group n = 3. Wt, wild-type; Mut, mutant; miR, microRNA; NC, normal cells; OE, overexpressing MiR-30a-5p cells.

The TargetScan database analysis identified BCL2L11 as the miR-30a-5p target site (Figure 8A). To examine the interaction between miR-30a-5p and its target site in BCL2L11 mRNA, a luciferase reporter gene assay was performed using constructs containing the predicted target sequence (BCL2L11-wt) and the mutated target sequence (BCL2L11-mut). We found that cotransfection of miR-30a-5p mimics and BCL2L11-wt in BCSCs resulted in decreased luciferase activity compared to the scrambled control, whereas cotransfection of miR-30a-5p mimics and BCL2L11-mut in BCSCs showed luciferase activity comparable to the scrambled control (Figure 8B). Overexpression of miR-30a-5p significantly reduced the proportion of late apoptotic cells induced by RA and also slightly reduced the proportion of total apoptotic cells (all *P* < 0.05) (Figures 8C, D). These results suggest that miR-30a-5p binds to BCL2L11 and regulates apoptosis.

## 4 Discussion

Breast cancer is the most commonly diagnosed cancer in women, and ranks second among the causes of cancer-related death in women (Fahad, 2019). Triple-negative breast cancer, which has a high invasive nature, poor prognosis, high recurrence rate, and high mortality rate, accounts for about 15%–20% of all breast cancer pathologic types (Ueda et al., 2020; Yin et al., 2020). Breast cancer transformed into stem cells has a strong ability to evade chemotherapy, and therefore such cancers are highly recurrent (Pan et al., 2024). We conducted cytotoxicity studies previously and found that the inhibitory effect of RA on breast tumor cells was much higher than that on ordinary breast cells (e.g., MCF-10A) (Ni et al., 2019). In addition, recent studies have found that RA has anti-tumor and other pharmacological effects (Nunes et al., 2017; Guan et al., 2022; Zhao et al., 2022; Ijaz et al., 2023), suggesting that RA may provides a selective therapeutic pathway of TNBC (Wei et al., 2020).

The results showed that CCK-8 assay, wound-healing assay, colony integration assay, and cell cycle assay provided strong evidence that RA inhibited the viability and migration of BCSCs, inhibited colony formation, and arrested cell cycle in the G0-G1 phase. This is consistent with our previous findings (Li et al., 2018a; Li et al., 2018b), and this anti-breast cancer effect (by promoting apoptosis) is recognized by other investigators (Scheckel et al., 2008; Stanojkovic et al., 2010).

In Figure 2, it was observed that BCSCs showed a significant inhibitory effect on cell aggregation after 50 μg/mL RA treatment in the cell cloning assay. In reality, the bioavailability of RA-rich herbal drugs, whether orally or intravenously, is not high, and the results of the *in vivo* pharmacokinetics in humans show that RA can reach *in vivo* blood concentrations of up to 142.20 ± 45.20 nmol/L and 308 ± 77 ng/mL (Baba et al., 2005; Jia et al., 2010; Hitl et al., 2021), In contrast, high accumulation is only possible in the kidney tissue of rats (Li et al., 2007). However, there were significant clinical efficacies at these blood concentrations, including antitumor effects, and thus the tumor-suppressive effects of low-dose RA may be multi-pathway, possibly interfering with the population dependence of cancer cells. In the present study, because of limited resources, we were temporarily unable to go further to explore the long-term benefits of RA at low doses in in vitro experiments, focusing on exploring the biological mechanisms of cancer inhibition at specific doses.

Apoptosis is a unique pattern of programmed cell death involving activation of a clear signaling cascade to eliminate cells. Apoptosis is also a key process for inhibiting tumor cell growth targeted by chemotherapeutic drugs. This study found that after 48 h of 200 μg/mL RA treatment, the apoptosis rate of BCSCs was significantly increased, while cell viability and cell scratches significantly decreased. Additionally, the colony formation assay showed that 100 μg/mL RA significantly inhibited cancer cell formation. This suggests that 200 μg/mL RA may be a more potent therapeutic concentration, with an irreversible induction of cell death. Therefore, in the following experiments, we focused on observing how RA within 200 μg/mL was used to initiate the apoptotic cascade effect.

Next, we observed changes at the molecular level in cell apoptosis-related proteins and miRNA levels. Consistent with our previous study, the BCL-2/Bax ratio decreased with increasing RA dose. At the same time, we detected the key apoptosis molecule p53 and the critical molecule BCL2L11. The regulatory direction further confirmed that RA promotes apoptosis in breast cancer cells.

Subsequently, we performed genome-wide methylation microarray analysis in BCSCs after RA treatment, and found that the methylation degree of the promoter region did not changed significantly, suggesting that this expression abnormality is likely caused by post-transcriptional regulation, and miRNA is one of the important mechanisms of post-transcriptional regulation.

Based on the above research background and previous studies, we believe that both miRNA and BCL-2/Bax pathway inhibit BCSCs in RA. Further investigation is needed to clarify whether RA regulates BCL-2/Bax gene expression in a coordinated manner. The literature evidence has shown that miRNA can regulate the two pathways, suggesting that it may be one of the potential front-end molecular regulation mechanisms. RA in Sarcandra glabra can affect the gene expression of BCL-2/Bax downstream by changing the miRNA levels in BCSCs, inducing apoptosis, and ultimately inhibiting BCSCs.

Micro-RNAs (miRNAs) are a class of small non-coding RNAs of 19–25 nucleotides in length (Winkler, 2010). Dysregulated tiny RNA may play a role in tumor suppression or promotion by targeting binding oncogenes or tumor suppressor genes (Zheng and Zhang, 2016). Studies show that miRNAs such as miR-27, miR-454, and those involved in MDA-MB-468 can affect proliferation, differentiation, metastasis, and invasion (Li Q. et al., 2017; Wu et al., 2018). miRNA functions in silencing mRNA or inhibiting its protein transcription. In this study, we detected differential miRNA expression in BCSCs by high-throughput sequencing, and through differential analysis and functional enrichment analysis to screen with apoptosis-related pathways, we identified the key miRNA, miR-30a-5p. MiR-30a-5p is a key regulatory molecule in breast tumors, and it inhibited breast cancer cell migration, invasion, and glycolysis by targeting MTDH (3′-untranslated region of metadherin) and LDHA (Lactate dehydrogenase A) (Zhang et al., 2014; Li L. et al., 2017). In this study, we used targetscan to predict the target of miR-30a-5p and found that its target gene was apoptotic molecule BCL2L11. Later, we used a dual luciferase reporter system to verify the association of miR-30a-5p and BCL2L11. The results showed a significant interaction between the two. This result suggested that RA can effectively inhibit the silencing effect of miR-30a-5p on the BCL2L11 gene.

The regulation of apoptosis by miR-30a-5p is also evident in its interaction with the BCL-2 gene. Xu (Xu et al., 2017) and Jian (Jian et al., 2019) believe that miR-30a-5p induces apoptosis by targeting the silencing of BCL-2. Quan (Quan et al., 2019) also reported that the high level of miR-30a-5p induced decrease of BCL-2 protein levels and promoted the increase of Bax protein levels. However, recent reports have shown that knockdown of miR-30a-5p significantly increased Bax levels and decreased BCL-2 levels, as validated in hypoxia/reoxygenation models in cardiomyocytes and renal tubular epithelial cells (He et al., 2020; Liang et al., 2024). In this study, we observed that miR-30a-5p of BCSCs was downregulated by RA. This downregulation reduced the silencing effect of miR-30a-5p on the BCL2L11 gene and increased the expression of Bim, a translation product of the BCL2L11 gene. Bim bound to BCL-2 and promoted the progression of apoptosis. The regulation of the apoptotic pathway by miR-30a-5p may vary across different tissues. Additionally, we considered the molecules that RA regulates at the microRNA level. Quan (Quan et al., 2019), chromatin immunoprecipitation assays showed that miR-30a-5p expression wasincreased following binding of p53 to the promoter of MIR30A. This finding aligns closely with our study. This supports the view that miR-30a-5p plays an important role in the regulation of apoptosis.

Similarly, this regulation of apoptosis may also be induced based on inhibiting cellular autophagy levels, when simultaneous downregulation of two key molecules, ATG 5 and BCL-2, is observed when miR-30a-5p is overexpressed (Tan et al., 2023). Surprisingly, our target was the key molecule BCL-2/Bim in this study. From the perspective of autophagy, the downregulation of BCL2L11 can improve the death threshold of cancer cells through two mechanisms, thus promoting their drug resistance: the direct inactivation of apoptotic signaling cascade and the induction of cytoprotective autophagy (Dai and Grant, 2015).

This also suggests that RA is not only a drug that fights breast cancer by promoting apoptotic signaling, but also one that may reverse cancer cell resistance and acts in combination with other antitumor drugs. However, the detailed mechanisms need further investigation. In summary, RA treatment may exert its anti-tumor effects by modulating the highly relevant miR-30a-5P in BCSCs, offering new ideas for the diagnosis, treatment, and prognosis of TNBC.

## 5 Conclusion

RA effectively inhibited the silencing effect of miR-30a-5p on the BCL2L11 gene and enhanced apoptosis in BCSCs.

## Data Availability

The original contributions presented in the study are publicly available. This data can be found here: GEO repository (https://www.ncbi.nlm.nih.gov/geo/), accession number GSE274675.
