# Supplementary figures and images for: The role of the miR-30a-5p/BCL2L11 pathway in rosmarinic acid-induced apoptosis in MDA-MB-231-derived breast cancer stem-like cells
Source: Front Pharmacol. 2024 Aug 22;15:1445034. doi: 10.3389/fphar.2024.1445034 (PMC11375422; doi:10.3389/fphar.2024.1445034)

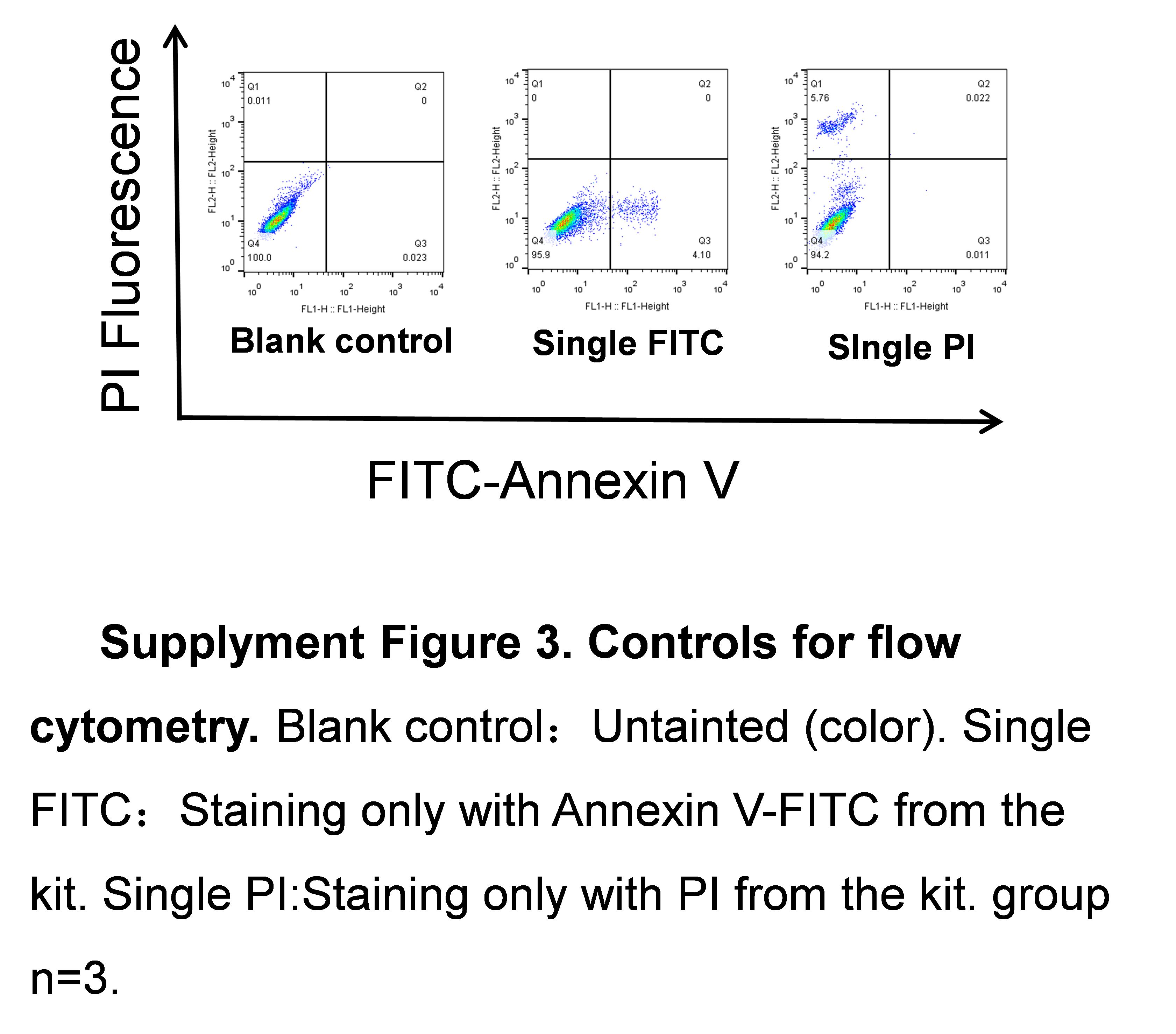

Supplement: Supplementary file 1 [file Image3.jpg]

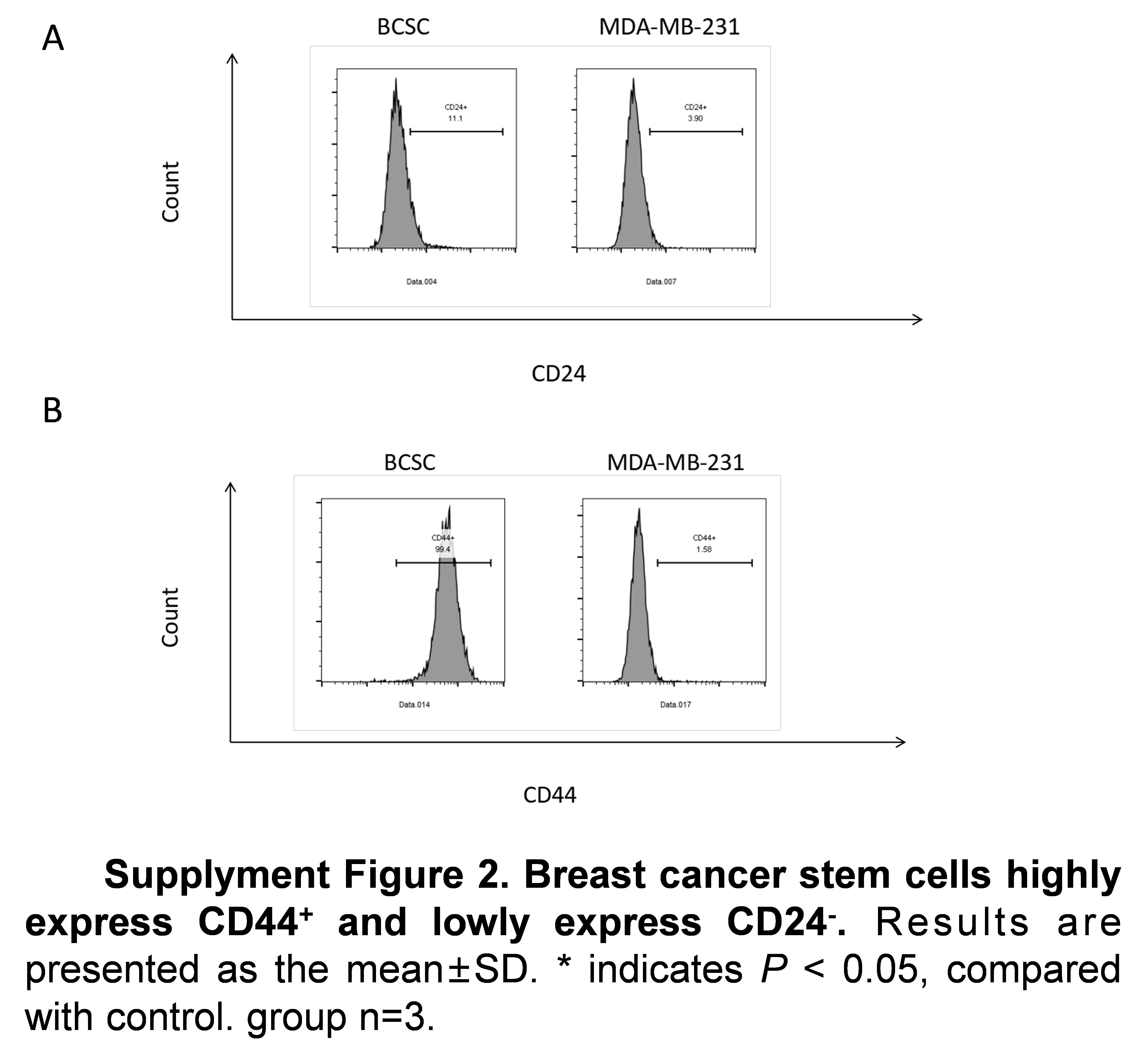

Supplement: Supplementary file 2 [file Image2.jpg]

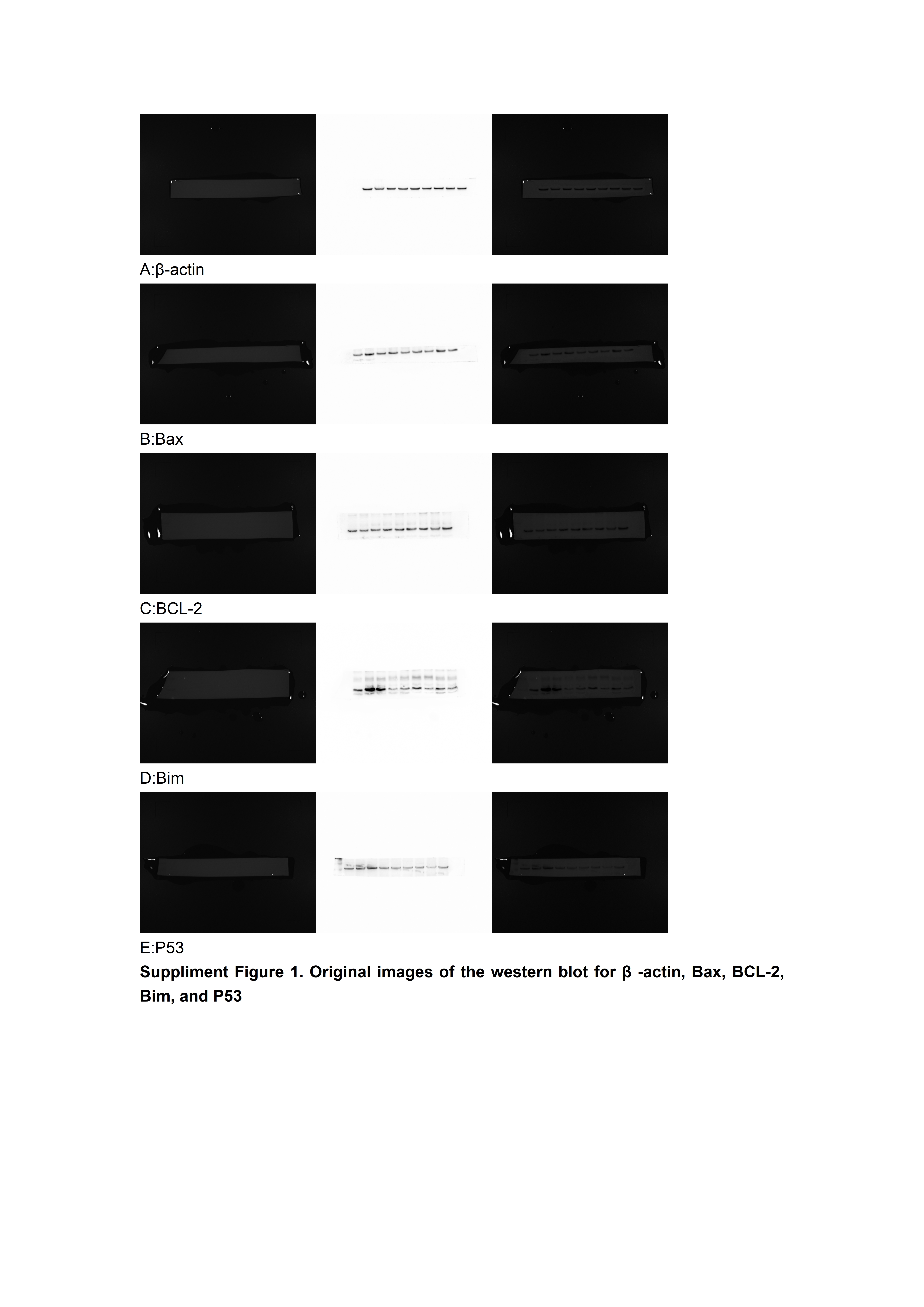

Supplement: Supplementary file 3 [file Image1.jpg]
